# Effect of thong style flip-flops on children’s midfoot motion during gait

**DOI:** 10.1186/1757-1146-5-S1-O19

**Published:** 2012-04-10

**Authors:** Angus Chard, Andrew Greene, Adrienne Hunt, Benedicte Vanwanseele, Richard Smith

**Affiliations:** 1 Exercise and Sport Science, University of Sydney, Sydney, NSW, 1825 Australia; 2 Department of Biomedical Kinesiology, Katholieke Universiteit Bus 5005 3000 Leuven, Belgium

## Background

Thong style flip-flop footwear (TH) and sandals are the preferred footwear of 22% of Australian children, however little is known about the effects of wearing TH on the growing child [1]. Previous research has shown TH reduces children’s hallux dorsiflexion prior to contact during walking and jogging and at toe-off whilst jogging [2]. Adult studies have shown TH alters barefoot motion with reduced eversion [3] and reduce peak plantar-pressure at the hallux, metatarsal heads and calcaneus [4].The influence of TH on children’s midfoot kinematics may have important ramifications for children’s developing feet. This study aims to describe the effect of TH on children’s midfoot motion during walking and jogging.

## Materials and methods

Seven healthy children, mean age 10.47±1.98 years were recruited from Sydney Australia. Participants conducted five walking trials and five jogging trials while barefoot and wearing TH in random order. A fourteen camera, three-dimensional motion analysis system was used to collect kinematic data. Markers located at navicular, first and fifth metatarsal phalangeal joints, hallux and a rearfoot wand, defined three foot segments: rearfoot, forefoot and hallux. The midfoot joint was defined as the articulation between rearfoot and forefoot segments.

## Results

**Figure 1:**
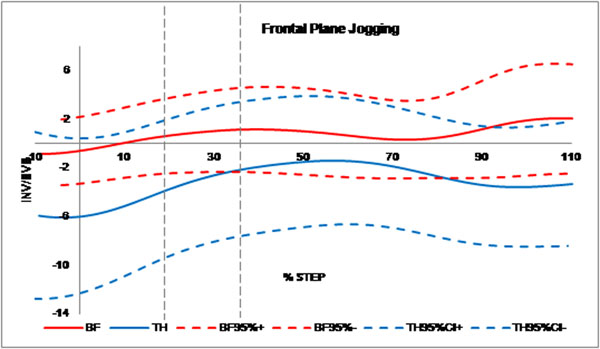
Mean midfoot frontal plane range of motion data from -10% to +10% of the stance phase of gait for barefoot ( red) and thongs (blue), including ± 95% CI. Contact, midstance and propulsion phases are defined by vertical dashed lines.

A repeated measure ANOVA found no significant effect of thongs while walking when compared to barefoot although a trend was seen towards a more dorsiflexed, everted and abducted midfoot. A significant effect of TH while jogging was seen during propulsion in the frontal plane (Figure 1) with the forefoot more inverted (P=0.016) in the TH condition -4.5±6.3SD compared with BF 3.8±5.0SD at toe-off, while sagittal and transverse planes were not significantly different.

## Conclusions

During the propulsive phase significantly greater forefoot inversion was seen while jogging wearing TH. Results reported are early findings of an ongoing project. With greater participant numbers the trends of greater forefoot extension, eversion and abduction while walking may become significant.
